# Water-soluble polyphosphonate-based bottlebrush copolymers *via* aqueous ring-opening metathesis polymerization†

**DOI:** 10.1039/d3sc02649c

**Published:** 2023-09-26

**Authors:** Diego A. Resendiz-Lara, Suna Azhdari, Hubert Gojzewski, Andre H. Gröschel, Frederik R. Wurm

**Affiliations:** a Sustainable Polymer Chemistry (SPC), Department of Molecules and Materials, MESA+ Institute for Nanotechnology, Faculty of Science and Technology, Universiteit Twente PO Box 217 7500 AE Enschede The Netherlands frederik.wurm@utwente.nl; b Physical Chemistry, University of Münster Corrensstraße 28–30 Münster 48149 Germany

## Abstract

Ring-opening metathesis polymerization (ROMP) is a versatile method for synthesizing complex macromolecules from various functional monomers. In this work, we report the synthesis of water-soluble and degradable bottlebrush polymers, based on polyphosphoesters (PPEs) *via* ROMP. First, PPE-macromonomers were synthesized *via* organocatalytic anionic ring-opening polymerization of 2-ethyl-2-oxo-1,3,2-dioxaphospholane using *N*-(hydroxyethyl)-*cis*-5-norbornene-*exo*-2,3-dicarboximide as the initiator and 1,8-diazabicyclo[5.4.0]undec-7-ene (DBU) as the catalyst. The resulting norbornene-based macromonomers had degrees of polymerization (DP_*n*_) ranging from 25 to 243 and narrow molar mass dispersity (*Đ* ≤ 1.10). Subsequently, these macromonomers were used in ROMP with the Grubbs 3^rd^-generation bispyridyl complex (Ru-G3) to produce a library of well-defined bottlebrush polymers. The ROMP was carried out either in dioxane or in aqueous conditions, resulting in well-defined and water-soluble bottlebrush PPEs. Furthermore, a two-step protocol was employed to synthesize double hydrophilic diblock bottlebrush copolymers *via* ROMP in water at neutral pH-values. This general protocol enabled the direct combination of PPEs with ROMP to synthesize well-defined bottlebrush polymers and block copolymers in water. Degradation of the PPE side chains was proven resulting in low molar mass degradation products only. The biocompatible and biodegradable nature of PPEs makes this pathway promising for designing novel biomedical drug carriers or viscosity modifiers, as well as many other potential applications.

## Abstract 2

Water-soluble and biodegradable polyphosphoester polymer bottlebrush copolymers and amphiphilic assemblies prepared by a combination of anionic ring-opening and metathesis polymerization.

## Graphical abstract

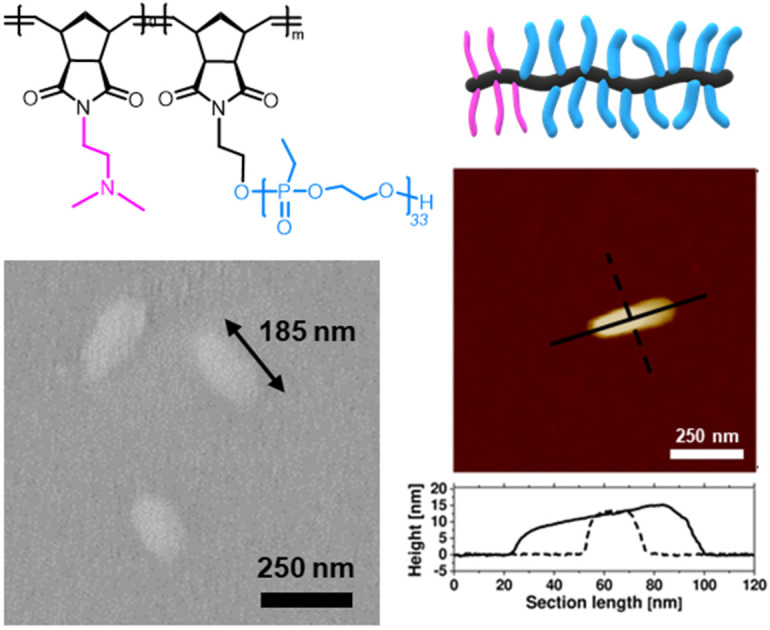

## Introduction

Bottle-brush polymers (BBPs) are macromolecules with complex topologies, in which flexible or rigid polymeric side-chains are attached to a linear backbone with promising properties for diverse applications, *e.g.* in lubrication, optoelectronic materials, super-soft elastomers, or drug delivery.^1–9^ With the well-known ruthenium-based metathesis, ring-opening metathesis polymerization (ROMP) operates with fast initiation and propagation, with controlled distributions and architectural control. These highly active catalysts tolerate a myriad of substrates and a range of reaction conditions, including air and moisture.^10–14^

Over the last decade, significant efforts to perform ROMP under aqueous conditions^15,16^ have been successful and different strategies have been developed. For instance, the use of water-soluble ROMP catalysts has facilitated the synthesis of complex macromolecular architectures in aqueous conditions; however, this requires the modification of the catalyst.^17–20^ Very recently, a monomer oil-in-water emulsified aqueous ROMP was developed with quantitative initiation and a high degree of polymerization, but with the use of chlorinated solvents as the oil phase.^21^ In contrast, the O'Reilly group reported aqueous ROMP using a mixed solvent system H_2_O/THF (v/v = 9/1) using Grubbs' third generation catalysts (Ru-G3).^22,23^ Aqueous ROMP might be applied to other water-soluble monomers,^24^ in the modification of biomacromolecules,^18,25,26^ or the formation of controlled nanoparticles through ROMP with a more sustainable approach.^22,27^

PPEs are well-defined, water-soluble polymers and potential alternatives to poly(ethylene glycol) (PEG) but with a controlled degradation pattern.^28^ The chemistry of PPEs is versatile, allowing hydrophilicity, thermo-responsiveness, and (bio)degradation rates to be tuned.^28–30^ PPEs are appealing for biological and biomedical applications due to their nucleic acid analogue structure, water-solubility, anti-biofouling properties, cytocompatibility, and the so-called ‘stealth effect’.^31–33^ Specifically, PPE-containing nanomaterials have been employed in surface protein adsorption,^34^ as drug or gene delivery nanocarriers,^35–37^ due to their antifouling properties and antimicrobial nanoparticles.^38^ Our efforts in the synthesis of PPEs have been focused on one subclass, *i.e.*, polyphosphonates [–P(O)R–OCH_2_CH_2_O–]_*n*_, which can be obtained with high control over molar mass (*M*_n_) and narrow distributions (*Đ* < 1.2) from the anionic ring-opening polymerization (AROP) of cyclic phosphonates (CH_2_O)_2_P(O)R.^31,39^ Especially, polyphosphonates (R = Me, Et, iPr, allyl) are water-soluble and are recognized as degradable PEG alternatives.^28,33^

**Scheme 1:**
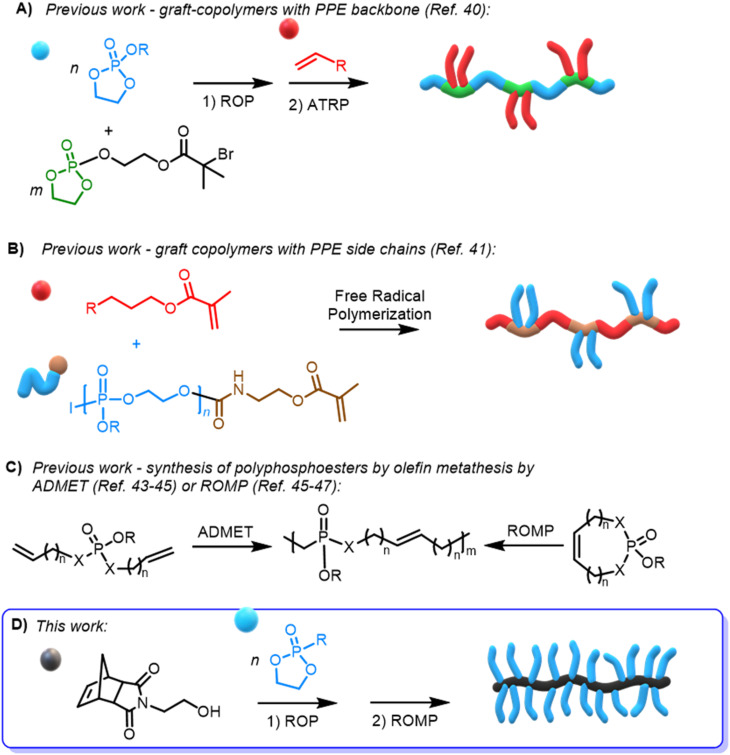
(A and B) Synthesis of graft-polyphosphoesters by (A) ATRP or (B) free-radical polymerization. (C) Synthesis of polyphosphoesters by ADMET or ROMP. (D) Preparation of norbornene-based polyphosphonate macromonomers and subsequent ROMP to bottlebrush polymers.

So far, only scarce reports on PPE-based graft polymers *via* atom transfer radical polymerization (ATRP)^40^ (Scheme 1A) or free radical polymerization^41^ (Scheme 1B) have been reported; however, materials with narrow molar mass dispersity remain challenging. The application of olefin metathesis polymerization of phosphate-based materials has been limited to post-functionalization of ROMP polymers with phosphonates^42^ or by acyclic diene metathesis (ADMET)^43–45^ or ROMP of 7-membered cyclic phosphoesters^45,46^ or phosphoramidates^47^ (Scheme 1C), generally resulting in low molar mass PPEs with uncontrolled distributions. Other phosphoester-based norbornene oligomers have been synthesized *via* ROMP and used as either benzylating^48^ or triazolating reagents.^49^

Concerns about PEG as a non-biodegradable polyether, which can accumulate in the body and lead to the formation of antiPEG-antibodies,^50–52^ have motivated the search for other bottlebrush type materials for biomedical applications derived from other functional hydrophilic polymers as PEG alternatives, *e.g.* norbornene-based polyoxazolines^53^ or *N*-carboxyanhydrides,^54,55^ but the application of ROMP of such macromonomers is neither under biorelevant conditions nor are the side chains prone to degrade.

In detail, we present PPE-based bottlebrush (co)polymers as a novel class of materials with high molar masses prepared by aqueous ROMP (Scheme 1D). We used the AROP of 2-ethyl-2-oxo-1,3,2-dioxaphospholane (*i.e.*, ethyl ethylene phosphonate, EtPn) initiated by *N*-(hydroxyethyl)-*cis*-5-norbornene-*exo*-2,3-dicarboximide to prepare a library of well-defined and water-soluble poly(ethyl ethylene phosphonate (PEEP)-macromonomers for ROMP with DP_*n*_ between *ca.* 20 and 300. These macromonomers were applied in a ‘grafting through’ ROMP to well-defined BBPs bearing polyphosphonates as the side chains. The ROMP of the PPE-macromonomers was performed in dioxane or in aqueous conditions without the need for catalyst modifications relying solely on the Grubbs 3^rd^-generation catalyst (Scheme 1D). Control over the length of the side-chains and the BBP backbone was independently achieved using polyphosphonate macromonomers with different DP_*n*_ and changing the macromonomer to catalyst ratio, respectively. In addition, a two-step approach was used to prepare double hydrophilic diblock copolymers using sequential ROMP: first by polymerization of a hydrophilic monomer in a water-soluble organic solvent (THF) and then by using the living chain as a macroinitiator in neutral aqueous media to chain-extend with the PPE-macromonomers.

## Results and discussion

Norbornene-functionalized polyphosphonate macromonomers (NB-PEtPn) were synthesized *via* organocatalytic AROP of 2-ethyl-2-oxo-1,3,2-dioxaphospholane (1, EtPn) using *N*-(hydroxyethyl)-*cis*-5-norbornene-*exo*-2,3-dicarboximide (2) as the initiator. The AROP was conducted in dichloromethane at room temperature with 1,8-diazabicyclo[5.4.0]-undec-7-ene (DBU) as the catalyst (Scheme 1A).

**Fig. 1:**
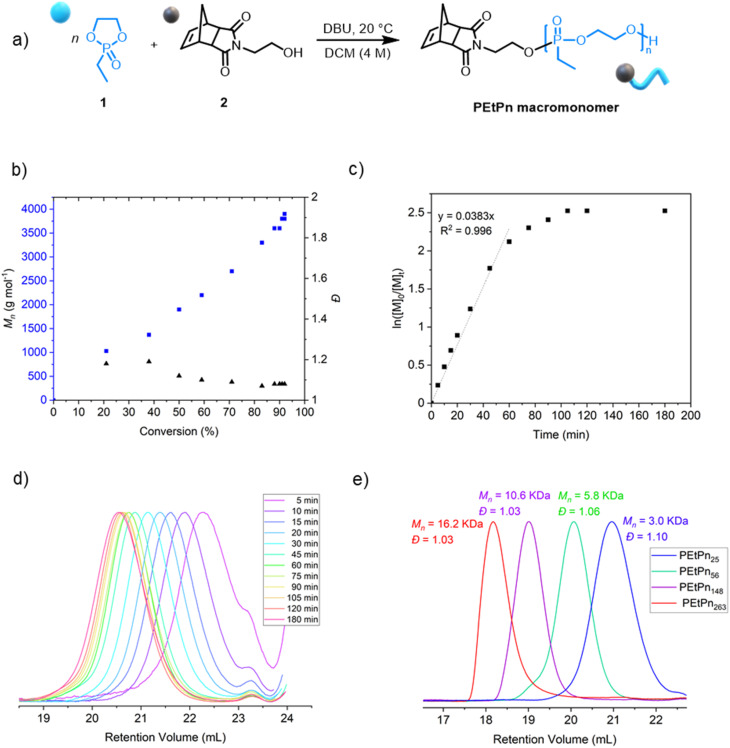
(a) Synthetic scheme for the AROP of PEEP (1) by *N*-(hydroxyethyl)-*cis*-5-norbornene-*exo*-2,3-dicarboximide (2) as the initiator. (b) Plot of *M*_n_ and *Đ vs.* monomer conversion, obtained by a combination of SEC and ^31^P NMR spectroscopy. (c) Kinetic studies of AROP of 1*via* the plot of ln([*M*]_0_/[*M*]_*t*_) *vs.* time. (d) SEC elugrams (2 mg mL^−1^) (normalized RI) at different reaction times for the polymerization of 1 to the PEtPn macromonomer (measured in DMF (0.1 M LiCl) at 60 °C *vs.* polystyrene standards using an RI detector by SEC). (e) SEC elugrams (2 mg mL^−1^) (normalized RI) of isolated PEtPn macromonomers with different degrees of polymerization DP_*n*_ = 25 (blue, *Đ* = 1.10), DP_*n*_ = 56 (green, *Đ* = 1.06), DP_*n*_ = 148 (green, *Đ* = 1.03) and DP_*n*_ = 263 (red, *Đ* = 1.03) (measured in DMF (0.1 M LiCl) at 60 °C *vs.* polystyrene standards using the RI detector by SEC).

The kinetics of the AROP of 1 with the initiator 2 was assessed (Table S2†). Similar to earlier reports using other functional initiators,^39,56^ high control over the molar mass and molar mass dispersity (*Đ* < 1.10) was achieved under the experimental conditions, reaching 92% conversion after *ca.* 105 min (Fig. 1b). The linear relationship between the increase of the molar mass *M*_n_ and the monomer conversion as well as a linear relation of ln([*M*]_0_/[*M*]_*t*_) *versus* time indicated a living polymerization using the norbornene imide-based initiator (Fig. 1c and d). A library of well-defined PPE-macromonomers with degrees of polymerization (DP_*n*_) between 20 and 300 was prepared (Fig. 1e and Table S1†). The polymers were obtained as colorless and viscous materials at room temperature, typical for PEEP. The presence of the initiator and the absolute molar mass were also retrieved by ^1^H NMR (Fig. S1†).

**Fig. 2:**
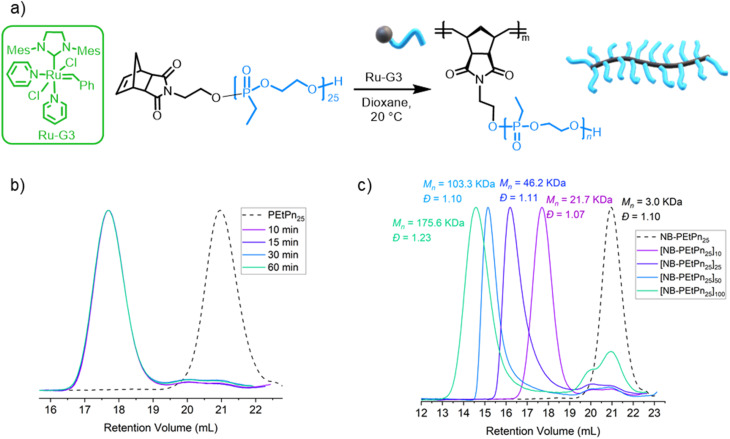
(a) Synthetic scheme of the graft-through ROMP of 3 using the Grubbs 3^rd^-generation complex (Ru-G3). (b) SEC elugrams (2 mg mL^−1^) (normalized RI) at different reaction times for the polymerization of ROMP of norbornene-based PEtPn_25_ using the Ru-G3 catalyst with varying PEtPn_25_ : Ru-G3 ratios of 10 : 1 (measured in DMF (0.1 M LiCl) at 60 °C *vs.* polystyrene standards using the RI detector by SEC). (c) SEC elugrams (2 mg mL^−1^) (normalized RI) of polymers from ROMP of norbornene-based PEtPn_25_ using the Ru-G3 catalyst with varying PEtPn_25_ : Ru-G3 ratios (10, 25, 50 and 100) (measured in DMF (0.1 M LiCl) at 60 °C *vs.* polystyrene standards using the RI detector by SEC).

The grafting through polymerization of the NB-PEtPn macromonomers was investigated in dioxane first (Fig. 2a). NB-PEtPn_25_ was dissolved in dioxane (0.05 M) followed by addition of the Ru-G3 catalyst ([NB-PEtPn_25_] : [Ru-G3] ratio; *i.e.* DP_*n*_ = 10 : 1) at 22 °C. To monitor the reaction progress, samples of the reaction were taken at different time points (10, 15, 30, and 60 min) and terminated by the addition of excess ethyl vinyl ether (EVE). SEC analysis of these samples revealed that full conversion was achieved to form a polyphosphonate BBP P(NB-*g*-PEtPn_25_)_10_ in only 10 min under these conditions (Fig. 2b). After the successful preparation of the first BBP, different ratios of [NB-PEtPn_25_] : [Ru-G3] between 10, 25, 50 and 100 equivalents were reacted under the same conditions (Fig. 2c and S6†). SEC analyses showed high conversion (*ca.* 90%) in all cases with a narrow molar mass dispersity of *Đ* < 1.11, except for the largest [NB-PEtPn_25_] : [Ru-G3] ratio (Table S3†). The ^1^H NMR spectra of the resulting BBPs showed the characteristic resonances for the polyphosphonate at 4.25, 1.84 and 1.21 ppm and the typical resonances of the polynorbornene imide backbone at 5.75 and 5.53 ppm, while the resonance of the initial norbornene alkene of the NB-PEtPn macromonomer at 6.29 ppm was no longer visible (Fig. S4†). The ^31^P NMR spectra show the same resonance for the polyphosphonate macromonomer and the BBP at *ca.* 35.2 ppm (Fig. S5†).

Besides changing the DP_*n*_ of the polymer backbone, the DP_*n*_ of the side chains was also varied by grafting through ROMP of the other NB-PEtPn macromonomers between 20 and 300 repeating units (Table S4†). We used a [NB-PEtPn_*n*_] : [Ru-G3] ratio of 25 : 1 at 22 °C in dioxane (0.05 M). The reactions were terminated after 24 h by addition of EVE. SEC analyses revealed that the NB-PEtPn conversion to P(NB-*g*-PEtPn_25_)_*n*_ decreased with an increase in the macromonomer DP_*n*_. For example, the conversion decreased from *ca.* 90% to 75% and *ca.* 60% when macromonomers with 25, 75 and 148 repeating units were used, respectively (Fig. S7†). For the highest DP_*n*_, mainly unreacted macromonomers and low molar mass bottlebrush polymers were observed even after 6 days of reaction (Fig. S8†). The latter observation is in agreement when increasing the macromonomers' degree of polymerization the conversion decreased, as a consequence of the low concentration of the chain-end polymerizable moiety and the additional steric hindrance of side chains of macromonomers.^57^ Another explanation might be the decreased initiation efficiency due to catalyst decomposition caused by water at a low concentration of the catalyst.^23^

**Fig. 3:**
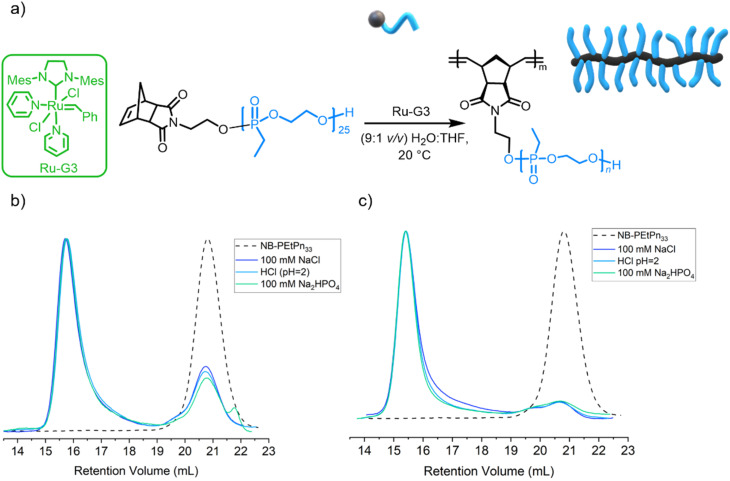
(a) Synthetic scheme for the graft-through ROMP of norbornene-based PEtPn_33_ using the Ru-G3 catalyst under aqueous conditions (9 : 1 v/v H_2_O/THF). (b) Overlay of SEC elugrams (normalized RI) of the polymerization of norbornene-based PEtPn_33_ using the Ru-G3 catalyst under different aqueous reaction conditions at a concentration of 10 mg mL^−1^ after 2 h and (c) at a concentration of 20 mg mL^−1^ after 2 h (measured in DMF (0.1 M LiCl) at 60 °C *vs.* polystyrene standards using the RI detector by SEC).

As PEEP is a water-soluble polymer with a similar partition coefficient (log *P*) value to PEG,^33^ the grafting through the ROMP process of NB-PEtPn was investigated in aqueous conditions. The O'Reilly group had successfully reported ROMP in water under different aqueous conditions using Ru-G3 as a catalyst^23^ and demonstrated the influence of solution pH and the presence of salt additives on the catalytic activity and stability during polymerization. In this work, we explored those reaction conditions for the formation of BBPs using our macromonomers NB-PEtPn in water (Fig. 3a and Table 1). First, acidic conditions (9 : 1 v/v H_2_O/THF, 100 mM Na_2_HPO_4_) were adjusted to a final pH = 2 using HCl and a concentration of 10 mg mL^−1^ was explored. Under these conditions, our initial attempts started with a NB-PEtPn_33_ and Ru-G3 catalyst at a [NB-PEtPn_33_] : [Ru-G3] ratio of 50 : 1. The polymerization was terminated after 2 h by the addition of one drop of diethylene glycol vinyl ether (DGVE), a water-soluble vinyl ether capable of deactivating the carbene propagating species. SEC analysis revealed a conversion of *ca.* 75% to P(NB-*g*-PEtPn_25_)_*n*_ (Fig. 3b). In order to simplify the reaction media, the sole use of HCl as a Brønsted acid additive was explored. The source of H^+^ and Cl^−^ as primary determinants for the metathetical activity in aqueous conditions has been proven to promote ligand dissociation and add further protection to the Ru-G3 catalyst from decomposition.^23^ We attempt the polymerization of NB-PEtPn_33_ and Ru-G3 at a [NB-PEtPn_33_] : [Ru-G3] ratio of 50 : 1 in a mixed solution (9 : 1 v/v H_2_O/THF) with pH = 2 (final concentration 10 mg mL^−1^), using only HCl as the acidifier additive. In the same manner, the reaction was terminated after 2 h of polymerization by DGVE and SEC analysis revealed a similar conversion (*ca.* 73%) to P(NB-*g*-PEtPn_33_)_*n*_ to the previous experiment (Fig. 3b). It is important to note that polyphosphoesters are stable at acidic pH for at least several hours as reported earlier.^58^ The role of the Cl^−^ anion has been determined, which avoids the formation of inactive Ru–(OH)_*n*_ species by ligand exchange by simply adding NaCl to the aqueous media.^23^ Finally, we attempted the ROMP at neutral pH, and the polymerization at a [NB-PEtPn_33_] : [Ru-G3] ratio of 50 : 1 was attempted in an aqueous solution (9 : 1 v/v H_2_O/THF, 100 mM of NaCl) with a final concentration of 10 mg mL^−1^. After 2 h of polymerization, the reaction was terminated by DGVE and the SEC analyses also revealed a similar conversion (72%) to the molecular brush P(NB-*g*-PEtPn_33_)_*n*_ (Fig. 3b). In general, the dispersity of the resultant BBPs was low (*Đ* < 1.1), which demonstrates control over ROMP under these aqueous conditions.

**Table 1:** ROMP of NB-PEtPn_33_ with Ru-G3 (50 : 1 molar ratio) in different aqueous conditions

Entry	Aqueous conditions	Concentration of NB-PEtPn_33_	Reaction time	Conversion^a^ (%)	*M*_n SEC_ b (kDa)	*Đ* b
1	100 mM Na_2_HPO_4_; pH = 2 (HCl)	10 mg mL^−1^	18 h	75	97.3	1.07
2	pH = 2 (HCl)	10 mg mL^−1^	18 h	74	89.2	1.10
3	100 mM NaCl	10 mg mL^−1^	18 h	71	93.7	1.09
4	100 mM Na_2_HPO_4_; pH = 2 (HCl)	20 mg mL^−1^	2 h	85	94.8	1.07
5	pH = 2 (HCl)	20 mg mL^−1^	2 h	88	91.7	1.07
6	100 mM NaCl	20 mg mL^−1^	2 h	90	88.9	1.09

a Conversion of NB-PEtPn_33_ to brush polymers is determined by integration of the peak areas of the brush polymer and residual NB-PEtPn_33_ from SEC measurement of the crude product.

b Determined from integration of the SEC signals of the high molar mass fraction (measured in DMF (0.1 M LiCl) at 60 °C *vs.* polystyrene standards using the RI detector by conventional SEC).

The three previous aqueous reaction conditions were successful in polymerizing the macroinitiator; however, they did not achieve complete conversion after 2 h at a concentration of 10 mg mL^−1^. To improve conversion to P(NB-*g*-PEtPn_33_)_*n*_, the reaction was monitored after 3 h and 18 h at 10 mg mL^−1^, by which the conversion increased from *ca.* 75% to *ca.* 85%, respectively, as observed by SEC analysis (Fig. S9†). We further attempted to improve the conversion to P(NB-*g*-PEtPn_33_)_*n*_ by increasing the initial concentration of the macromonomer to 20 mg mL^−1^. In this case, we achieved high conversion (*ca.* 90%) after only 2 h, as observed by SEC analysis (Fig. 3c).

**Fig. 4:**
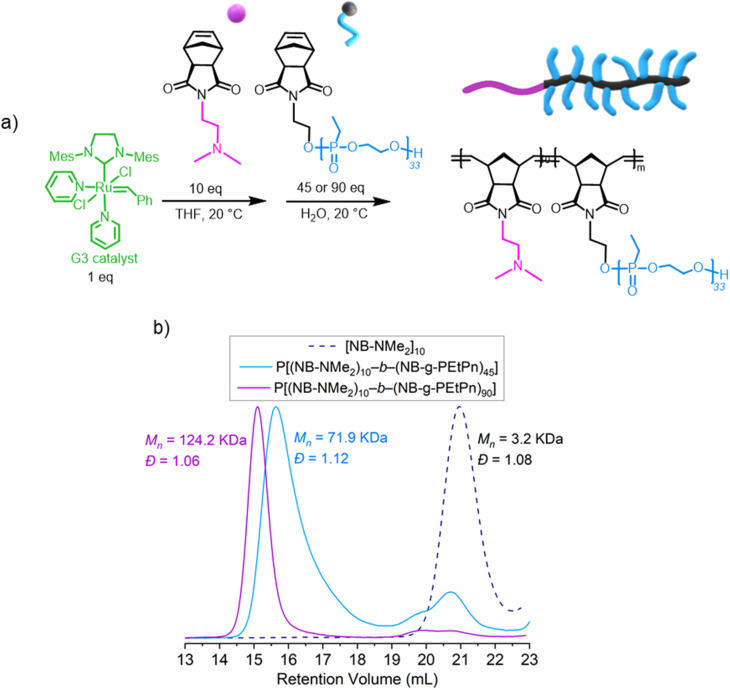
(a) Synthetic scheme for the sequential ROMP for the formation of block copolymers using the Ru-G3 catalyst. (b) SEC elugrams (normalized RI) of the polymerization of the sequential polymerization of [NB-NMe_2_] (DP_*n*_ = 10) and target of the second forming block (DP_*n*_ = 45 or 90) NB-PEtPn_33_ at a concentration of 20 mg mL^−1^ using the Ru-G3 catalyst under neutral aqueous conditions (9 : 1 v/v H_2_O/THF) 100 mM NaCl after 2 h (measured in DMF (0.1 M LiCl) at 60 °C *vs.* polystyrene standards using the RI detector by SEC).

We have successfully proved that the formation of molecular brush P(NB-*g*-PEtPn)_*n*_ at neutral aqueous conditions is possible. Encouraged by this, we explored the synthesis of diblock bottlebrush copolymers by a two-step approach in aqueous solution using Ru-G3 as a catalyst. First, polymerization of a few units of a hydrophilic monomer, *i.e. exo*-norbornene-2-(dimethylamino)ethyl imide (NB-NMe_2_), in a water-miscible organic solvent is prepared using Ru-G3. Then, the resulting corresponding macroinitiator can subsequently propagate in solvent mixtures containing high concentrations of H_2_O (*e.g.*, ≥90 v/v%) for chain-extension of a second monomer dissolved in water (Fig. 4a).

As the initial monomer to form the hydrophilic macroinitiator, the polymerization of NB-NMe_2_ at a [NB-NMe_2_] : [Ru-G3] ratio of 10 : 1 was first conducted in THF. Then, different amounts of the as-prepared macroinitiator were added to NB-PEtPn_33_ solutions (100 mM NaCl, neutral pH) to achieve different chain lengths of this second forming block (DP_*n*_ = 45 or 90) to evaluate the control over the polymerization. In this manner, the synthesis of two double hydrophilic P[(NB-NMe_2_)_*n*_-*b*-(NB-*g*-PEtPn)_*n*_] diblock copolymers was achieved with low dispersities (*Đ* < 1.12). High conversion (*ca.* 85%) was observed for P[(NB-NMe_2_)_10_-*b*-(NB-*g*-PEtP_33_)_45_], and high conversion (*ca.* 95%) was achieved for P[(NB-NMe_2_)_10_-*b*-(NB-*g*-PEtP_33_)_90_], as analyzed by SEC (Fig. 4b).

**Fig. 5:**
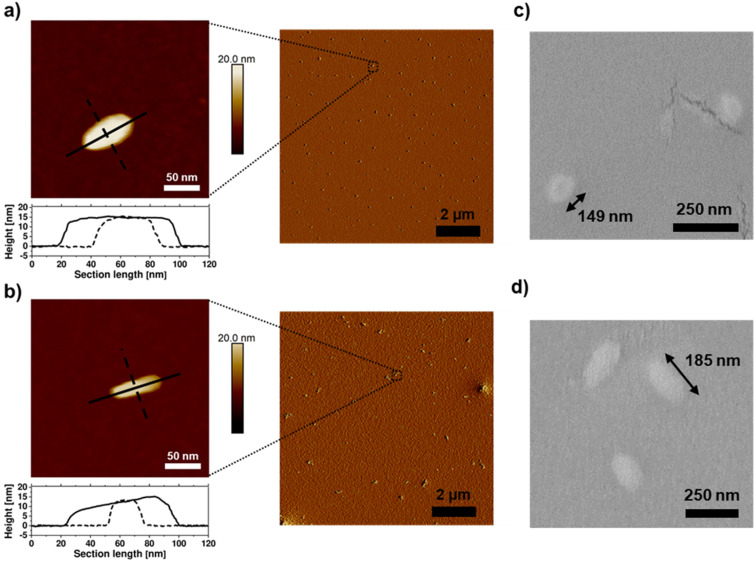
AFM height (left) and peakforce error (right) images of bottle brush block copolymers, (a) P[(NB-NMe_2_)_10_-*b*-(NB-*g*-PEtP_33_)_45_] and (b) P[(NB-NMe_2_)_10_-*b*-(NB-*g*-PEtP_33_)_90_], spin-cast at room temperature from an aqueous solution (10 mg mL^−1^), are shown. The cross-sections of the bottle brushes are plotted along the black lines. Additionally, SEM zoom-ins of (c) P[(NB-NMe_2_)_10_-*b*-(NB-*g*-PEtP_33_)_45_] and (d) P[(NB-NMe_2_)_10_-*b*-(NB-*g*-PEtP33)_90_] are presented, indicating the average length.

By atomic force microscopy (AFM), polymeric nano-objects with a rod-like morphology were observed after spin-coating aqueous solutions of the BBPs (10 mg mL^−1^). P[(NB-NMe_2_)_10_-*b*-(NB-*g*-PEtP_33_)_45_] exhibited anisotropic particles with an aspect ratio of approximately 2 : 1, while P[(NB-NMe_2_)_10_-*b*-(NB-*g*-PEtP_33_)_90_] showed objects with an aspect ratio of approximately 4 : 1 (the aqueous dispersion was spin-coated onto silicon wafers) (Fig. 5a and c). Anisotropic nanoparticles were in a similar size regime to other reports on BBPs.^59–62^ The anisotropy of the two BBP samples was also confirmed by SEM measurements. [(NB-NMe_2_)_10_-*b*-(NB-*g*-PEtP_33_)_45_] shows a roundish shape, whereby P[(NB-NMe_2_)_10_-*b*-(NB-*g*-PEtP_33_)_90_] clearly differs from this and is displayed as rods (Fig. S10a and b†). A size distribution of the particles was also determined which confirmed that the BBPs with DP_*n*_ = 45 have a length of 149 nm, and those with DP_*n*_ = 90 are slightly longer with 185 nm (Fig. 5b, d, S11a and b†). The height of 15 nm, determined by AFM, supports the assumption of densely grafted arms that prevent flattening due to steric hindrance (Fig. 5a and c). This observation highlights that the domains in the densely grafted BBPs exhibit significantly larger sizes and volumes compared to those of traditional block copolymers. This distinction holds considerable relevance for future self-assembly studies, as the increased volume and stiffness of BBPs, as opposed to BCPs, exert notable influence on self-assembly behavior. Consequently, the domain spacing in BBPs can be more readily adjusted, offering potential advantages for fine-tuning self-assembly processes. The correlated with the DLS measurements: measurements were conducted in DMF, indicating a hydrodynamic radius of 233 ± 4 nm for [(NB-NMe_2_)_10_-*b*-(NB-*g*-PEtP_33_)_45_] (Fig. S12†) and 312 ± 9 nm for P[(NB-NMe_2_)_10_-*b*-(NB-*g*-PEtP_33_)_90_] (Fig. S12†). It's worth noting that the diameters derived from DLS in DMF tend to be larger relative to those obtained *via* SEM and AFM due to swelling in dispersion.

In contrast to previously reported water-soluble BBPs, which have been studied in biomaterials, drug delivery, and encapsulation, the herein reported polyphosphoester-bearing BBPs carry degradable side chains.^63–66^ As proof of concept, P[(NB-NMe_2_)_10_-*b*-(NB-*g*-PEtP_33_)_45_] was stirred in a borate buffer solution (pH = 11) at 25 °C (5 mg mL^−1^) for a period of 24 h and the degradation product was analyzed by ^1^H NMR spectroscopy. The ^1^H NMR spectrum showed complete degradation of the polyphosphoester side chains as evidenced by the absence of resonances at 4.25, 1.84 and 1.21 ppm, which correspond to the main chain and side chain signals of the polyphosphoesters, respectively. Also in ^31^P NMR spectroscopy spectra, the absence of the PPE-backbone resonance at 35 ppm further evidenced the degradation of the side chains (Fig. S13†). SEC analysis showed the hydrolytic process of the polymeric side chain in the BBP by decreasing the *M*_n_ from *ca.* 71 kg mol^−1^ to less than 1 kg mol^−1^ (Fig. S14†). The degradation pathway of the side chain polyphosphoesters likely follows the backbiting mechanism as previously demonstrated for other polyphosphonates.^67^ As the backbone of the herein prepared BBPs cannot further degrade, in future studies additional labile bonds can also be installed, similar to previously reported studies for linear ROMP-based polymers.^47,68,69^

## Conclusion

Water-soluble PEtPn macromonomers (P1) carrying a polymerizable norbornene group were synthesized using AROP of 1 initiated by 2. These macromonomers were successfully used in ROMP to produce BBPs with precise two-dimensional control on the side-chain and in the main-chain backbone. The Grubbs 3^rd^-generation Ru-based catalyst was used in polar media to achieve high conversions and narrow molar mass distributions. The hydrophilicity of the macromonomers allowed for the synthesis of well-defined bottlebrushes under aqueous conditions, both under acidic and neutral aqueous conditions, *via* ROMP. A two-step approach enabled the formation of well-defined double hydrophilic block copolymers. Future work is directed towards a PISA approach for the synthesis of degradable nanoparticles. This protocol provides an efficient and green alternative for producing bottlebrushes with degradable materials in aqueous media, using commercially available Ru-based metathesis catalysts. The results of this study set the foundation for future research using PPEs as biodegradable substitutes for PEG-based polymer bottlebrushes.

With the growing need for sustainable and biodegradable polymers, the development of PPE-based bottlebrushes using ROMP might have the potential to pave the way for the design of innovative materials with tunable properties, offering exciting possibilities for applications in fields ranging from biomedicine to nanotechnology.

## Author contributions

D. A. R.-L. carried out the polymer synthesis and analyzed the experimental results. S. A. performed SEM and DLS measurements and analysis and edited the manuscript. H. G. performed surface characterization and analysis and edited the manuscript. The manuscript was written and edited by D. A. R.-L., A. H. G., and F. R. W. The financial support acquired for the project leading to this publication was secured by F. R. W. and A. H. G.

## Conflicts of interest

There are no conflicts to declare.

## Supplementary Material

SC-014-D3SC02649C-s001
