# Supplementary material for: Water-soluble polyphosphonate-based bottlebrush copolymers via aqueous ring-opening metathesis polymerization
Source: Chem Sci. 2023 Sep 26;14(40):11273–82. doi: 10.1039/d3sc02649c (PMC10583743; doi:10.1039/d3sc02649c)
Supplement: SC-014-D3SC02649C-s001 [file SC-014-D3SC02649C-s001.pdf]

Electronic Supplementary Material (ESI) for Chemical Science.
This journal is © The Royal Society of Chemistry 2023

Supplementary Information for:

Water-Soluble Polyphosphonate-Based Bottlebrush Copolymers via Aqueous Ring-Opening Metathesis Polymerization

Diego A. Resendiz–Lara, [1] Suna Azhdari[1,2], Hubert Gojzewski,[1] Andre H. Gröschel,[2] and Frederik R. Wurm*,[1]

[1] Sustainable Polymer Chemistry (SPC), Department of Molecules and Materials, MESA+ Institute for Nanotechnology, Faculty of Science and Technology, Universiteit Twente, PO Box 217, 7500 AE Enschede, The Netherlands.

[2] Physical Chemistry, University of Münster, Corrensstraße 28–30, Münster 48149, Germany

E-mail: frederik.wurm@utwente.nl

Materials and Methods

All manipulations involving air- and moisture-sensitive reagents were carried out either under an atmosphere of nitrogen gas using standard Schlenk techniques or under an atmosphere of nitrogen within an M. Braun glovebox MB-BL-01 maintained at <0.1 ppm of H_2O and <0.1 ppm of O_2. All solvents and chemicals were purchased from *Sigma Aldrich, Acros Organics, Fluka* or *Fisher Scientific* and used as received unless otherwise stated. Anhydrous deuterated chloroform (≥99.8 atom % D) were purchased from Sigma Aldrich and stored over activated molecular sieves (4 Å). Workup of polymeric materials was performed in air using lab-grade solvents. MilliQ water was purified from a MilliQ Advange A10 purification system (Millipore, Billerica, Ma, USA). 1,8-diazabycyclo[5.4.0]undec-7-ene (DBU, Sigma Aldrich, 98.0%) was distilled from CaH_2 and stored over molecular sieves (3 and 4 Å) under nitrogen at –40 °C. *N*-(hydroxyethyl)-*cis*-5-norbornene-*exo*-2,3-dicarboximide was synthesized from *cis*-5-norbornene-*exo*-2,3-dicarboxylic anhydride (Sigma Aldrich, 95.0%) and 2-aminoethanol (Sigma Aldrich, 98.0%) via a literature method[S1] and was under vacuum during 2 days. 2-ethyl-2-oxo-1,3,2-dioxaphospholane (ethyl ethylene phosphonate, EtPn) (≥99.0% determined by ^{31}P NMR spectroscopy analysis) was synthesized via a literature method[S2] and stored under N_2 at –40 °C. [1,3-Dimesityl-2-imidazolidinylidene]dichloro(phenylmethylene) bis(pyridine)ruthenium(II) (Grubbs 3rd-generation complex, Ru-G3) was prepared from Grubbs 2nd-generation (H_2IMes)(PCy_3)$(Cl)_2$Ru=CHPh) (Sigma Aldrich, Grubbs Catalyst M204, Umicore) and pyridine (Sigma Aldrich, 99.0%) via a literature method[S3] and was dried at reduced pressure overnight. Ethyl vinyl ether (EVE, Sigma Aldrich, 98%) and diethylene glycol vinyl ether (DGVE, Sigma Aldrich, 98%) were used as received. To remove the stabilizers from dioxane (Sigma Aldrich, ≥99.0%), the solvent was passed through basic alumina plugs in Pasteur pipettes before use.

Nuclear Magnetic Resonance (NMR) Spectroscopy

^{1}H, ^{31}P NMR spectra were measured on a 400 MHz *Bruker AVANCE III Nanobay with a BBFO probe* or a 600 MHz *Bruker AVANCE III system with a BBO probe.* The temperature was kept at 298 K during the measurements. As deuterated solvent $CDCl_3$ was used. *MestReNova 10.0.2* from *Mestrelab Research S.L.* was used for the analysis of all measured spectra. The spectra were calibrated against the solvent signal ($CDCl_3$: δH = 7.26 ppm)

Size Exclusion Chromatography (SEC)

SEC measurements were performed in DMF (0.1 M LiCl) at 60°C with an *Agilent Technologies 1260 Infinity II, PSS SECcurity system* at a flow rate of 1 mL min^{-1}. Each sample injection volume was 50 µL, performed by *Agilent 1260-ALS autosampler*. 2 GRAM columns (PSS) in series with dimensions of 8 × 300 mm^2, 10 µm particle size, and pore sizes of resp. 1000 and 30 Å, including a 10 µm GRAM guard column were employed. Calibration was carried out using polystyrene standards supplied by *Polymer Standards Service*. The SEC data were plotted using the software *OriginPro 8G* from *OriginLab Corporation*.

Atomic Force Microscopy (AFM)

The bottle brushes were deposited from a solution (0.2 mg mL^{-1}) on silicon wafers by spin-coating (2000 rpm, 60 s, room temperature). Silicon wafers were cleaned by ultrasonication in acetone (10 min) and Piranha solution treatment (20 min). AFM height and peakforce error images were obtained in air and at room temperature using a MultiMode 8 AFM instrument with a NanoScope V controller (Bruker) operated in the PeakForce Tapping mode. The ScanAsyst setting was set to "on" in order to apply optimized scanning parameters, particularly the feedback loop and the applied load (hundreds of pN), for imaging bottle brushes. The AFM data was collected following a sine-wave sample-tip trajectory with a frequency of 2 kHz and utilizing a peak-force amplitude value of 20-50 nm. Soft AFM cantilevers were chosen with a nominal spring constant of 0.4 N/m and a tip with a nominal radius of 2 nm (Bruker, ScanAsyst-Air). The AFM optical sensitivity (deflection sensitivity) was calculated based on the thermal tune method based on the nominal spring constant.[S4]

Scanning electron microscopy (SEM)

SEM measurements were performed on a cryo-field emission SEM equipped with an energy-selective detector for 16-bit image series acquisition with up to 40,000×50,000-pixel resolution and in lens, chamber. Samples for SEM measurements were prepared by putting one drop of an approximately 0.1 g L^{-1} sample dispersion on a mica wafer and dried for at least 6 h. The sputtering was prepared with a Quorum PP3010T-Cryo chamber with integrated Q150T-Es high-end sputter coater and an Au-Cd target layer of 4 nm was sputtered on the samples.

Dynamic light scattering (DLS)

The DLS measurements were carried out using an ALV/CGS-3 (ALV-LSE-5004 correlator) goniometer system with a He-Ne laser light source emitting at a wavelength (λ) of 633 nm, providing a power of 35 mW. The temperature of the sample was precisely maintained at 293.1 ± 0.2 K, and a minimum equilibration time of 15 minutes was observed before each measurement to minimize convection effects. Toluene was utilized as the matching bath, and the temperature was monitored using the built-in sensor of the goniometer system. For the angle-dependent measurements, observation angles (θ) ranging from 40° to 120° were employed. Particle size distributions were determined at an observation angle (θ) of 90°. To ensure accuracy and reliability, all measurements were performed in triplicate. The obtained correlation functions were subjected to analysis using the Cumulant and CONTIN methods, which were implemented using the ALV-Correlator Software (Version 3.0.5.9).

Synthetic Procedures

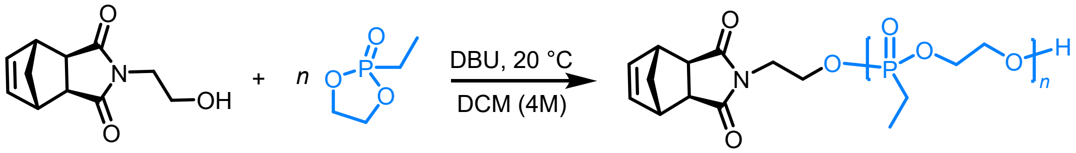

Scheme S1. Synthesis of *exo*-norbornene PEtn macromonomer by ring-opening polymerization of ethyl phosphonate by *N*-(Hydroxyethyl)-*cis*-5-norbornene-*exo*-2,3-dicarboximide as the initiator.

NOTE: During the course of this work different ***exo*-norbornene PEtn macromonomers** were prepared by varying the [initiator / monomer] ratio and different DP_n values were targeted (see Table S1), the DP_n was determined by end-group analysis by ^{1}H NMR spectroscopy.

Synthesis of *exo*-norbornene Petn macromonomers (Table S2, entry 3).

Ethyl ethylene phosphonate (1g, 7.35 mmol, 25 eq) and *N*-(Hydroxylethyl)-*cis*-5-norbornene-*exo*-2,3-dicarboximide (61 mg, 0.29 mmol, 1 eq) were dissolved in anhydrous CH_2Cl_2 (1.83 mL) in an oven-dried 4 mL vial equipped with a magnetic stirring bar. The reaction mixture was homogenized by stirring at 20 °C followed by the addition of DBU (132 µL, 134 mg, 0.88 mmol, 3eq) and the solution was stirred at room temperature for 2 h before the reaction mixture was quenched by the rapid addition of an excess of formic acid solution in CH_2Cl_2 (20 mg mL^{-1}). The crude product was purified by precipitation into cold diethyl ether (–28 °C) three times, and drying *in vacuo* to yield $PetPn_{26}$ *exo*-norbornene macromonomer as a colourless viscous liquid (1.02 g, 95 %). The molar mass was determined by end-group analysis ^{1}H NMR spectroscopy by comparing the integral of the $–CH_2$ signal of the CTA agent (6.26 ppm) with the backbone signal (4.25 ppm), similar to as a previous report.[S5]

Characterization of an *exo*-norbornene PEtn macromonomer

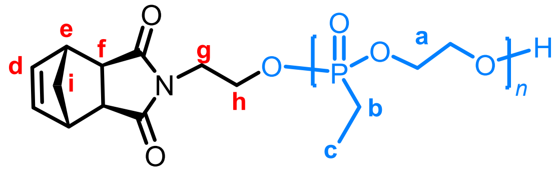

^{1}H NMR ($CDCl_3$): δ (ppm) 6.26 (t, J = 7.4 Hz, Hd, 2H); 4.31-4.09 (m, Ha); 4.00 (m, Hh, 2H), 3,24 (s, He, 2H); 2.87 (m, Hg, 2H); 2.71 (s, Hf, 2H); 1.49-1.31 (m, Hi, 2H); 1.86-1.67 (m, Hb), 1.19-1.10 (m, Hc). End-group ^{1}H NMR spectroscopy analysis showed DP_n = 26 based on 'd' (CH=CH). (Fig. S1)

^{31}P NMR ($CDCl_3$): δ (ppm) 35.2 (Fig. S2)

SEC: M_n = 3,000 g mol^{-1}, $Đ$ = 1.10 (Fig. S3)

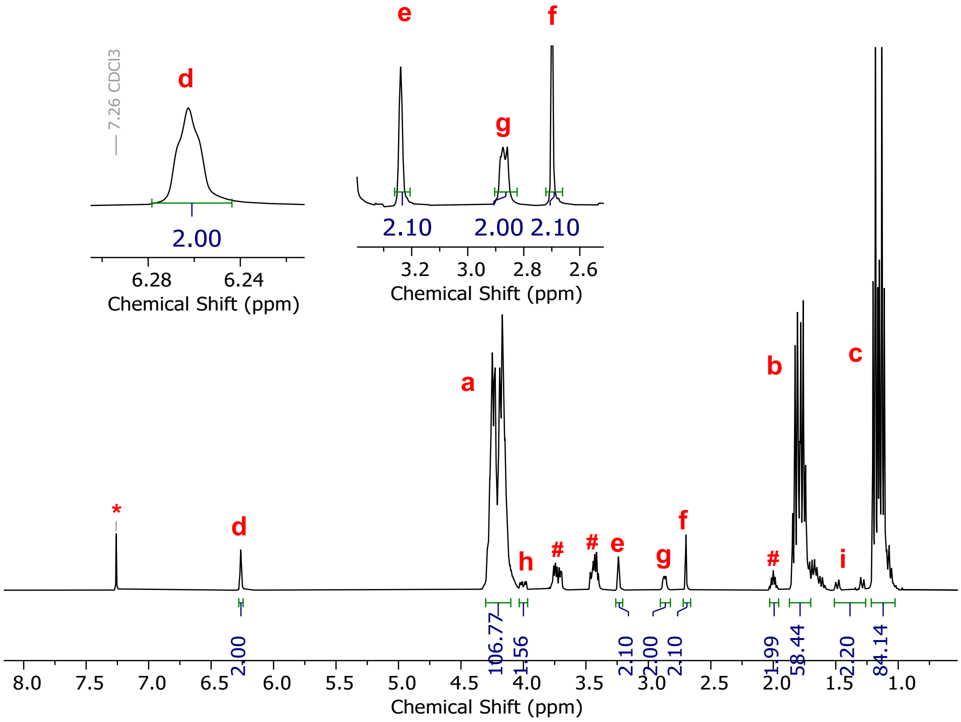

Figure S1**.** ^{1}H NMR (400 MHz, $CDCl_3$) spectrum of NB-PEtPn_{56}. Deuterated solvent residual signal denoted by * and end-groups of the phosphonate groups denote by #.

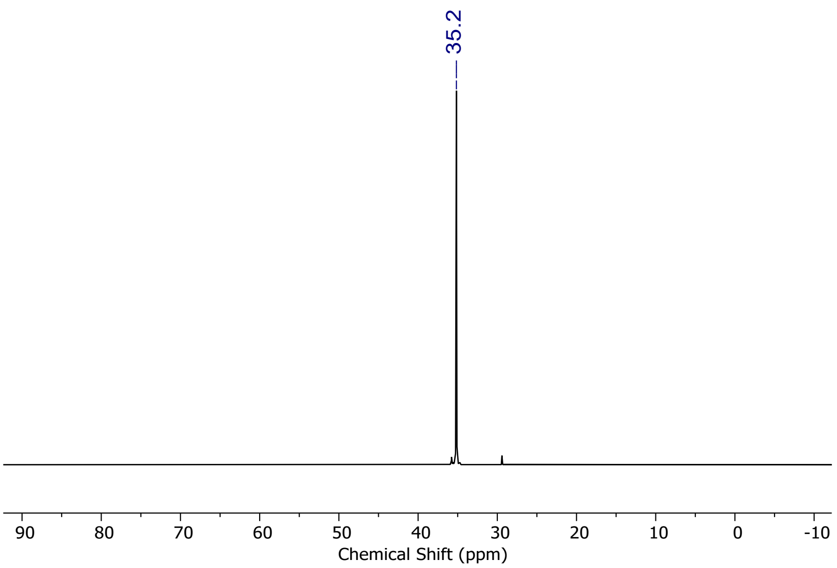

Figure S2. ^{31}P NMR (162 MHz, $CDCl_3$) spectrum of NB-PetPn$_{26}$.

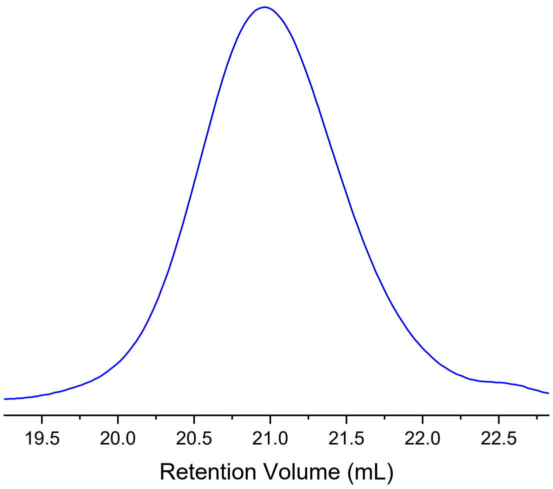

Figure S3. SEC elugram (2 mg mL^{-1}) (normalized RI) of isolated NB-PEtPn$_{26}$ (blue, *Đ*= 1.10) (measured in DMF (0.1 M LiCl) at 60 °C).

Table S1. Synthesis of NB-PEtPn macromonomers with different degrees of polymerization.

Entry	$[M]_{monomer}$: $[M]_{initiator}$	Yield (%)	$M_{n\ theo}$ [a]	$M_{n\ SEC}$ [c]	DP_n [b]	$Đ$ [c]
1	25:1	96	3,600	3,000	26	1.10
2	30:1	95	4,700	3,400	33	1.09
3	60:1	91	7,800	5,800	56	1.06
4	120:1	84	20,300	10,600	148	1.03
5	240:1	88	36,000	16,200	263	1.03

[a] [(DP_n*136.09) + 207].

[b] Calculated by ^{1}H NMR spectroscopy.

[c] Determined by SEC (measured in DMF (0.1 M LiCl) at 60 °C vs. polystyrene standards using RI detector by conventional SEC).

Kinetics of the polymerization of ethyl phosphonate to the *exo*-norbornene PEtn macromonomer.

Ethyl ethylene phosphonate (400 mg, 2.94 mmol, 50 eq) and *N*-(Hydroxyethyl)-*cis*-5-norbornene-*exo*-2,3-dicarboximide (12 mg, 0.059 mmol, 1 eq) were dissolved in anhydrous CH_2Cl_2 (0.74 mL) in an oven-dried 4 mL vial equipped with a magnetic stirring bar. The reaction mixture was homogenized by stirring at 20 °C followed by the addition of DBU (26 μL, 27 mg, 0.18 mmol, 3eq), and the solution was stirred at room temperature. The sampling involved periodic extraction of aliquots from the reaction mixture (terminated by the addition of one drop of formic acid solution in CH_2Cl_2 (20 mg mL^{-1})), followed by dissolution in $CDCl_3$, which is a good solvent for both ethyl ethylene phosphonate and the corresponding polymer. Each sample was analyzed by ^{1}H and ^{31}P NMR spectroscopy (the chemical shift of the cyclic monomer (52.5 ppm) to the corresponding linear phosphonic acid ester (35.1 ppm) was observed as previously reported[S2] and SEC in DMF (0.1 M LiCl) was conducted at 60 °C.

Table S2. Kinetics of the polymerization of ethyl ethylene phosphonate by *N*-(Hydroxyethyl)-*cis*-5-norbornene-*exo*-2,3-dicarboximide as the initiator to *exo*-norbornene PEtn macromonomer.

Entry	$[M]_{monomer}$: $[M]_{initator}$	Time (min)	Conv. (%) [a]	$M_{n\ theo}$ [b]	$M_{n\ SEC}$ [c]	$Đ$ [c]
1	50:1	5	21	1,600	1,030	1.18
2	50:1	10	38	2,800	1,370	1.19
3	50:1	15	50	3,600	1,900	1.12
4	50:1	20	59	4,200	2,200	1.10
5	50:1	30	71	5,000	2,700	1.09
6	50:1	45	83	5,800	3,300	1.07
7	50:1	60	88	6,200	3,600	1.08
8	50:1	75	90	6,300	3,600	1.08
9	50:1	90	91	6,400	3,800	1.08
10	50:1	105	92	6,500	3,800	1.08
11	50:1	120	92	6,500	3,900	1.08
12	50:1	180	92	6,500	4,000	1.10

[a] Calculated by ^{31}P NMR spectroscopy analysis.
[b] [((conversion $[M]_{EtPn}$/100*$[M]_{monomer}$: $[M]_{initiator}$)*136) + 207].
[c] Determined by SEC (measured in DMF (0.1 M LiCl) at 60 °C vs. polystyrene standards using RI detector by conventional SEC).

Synthesis of bottle-brush polymers in dioxane

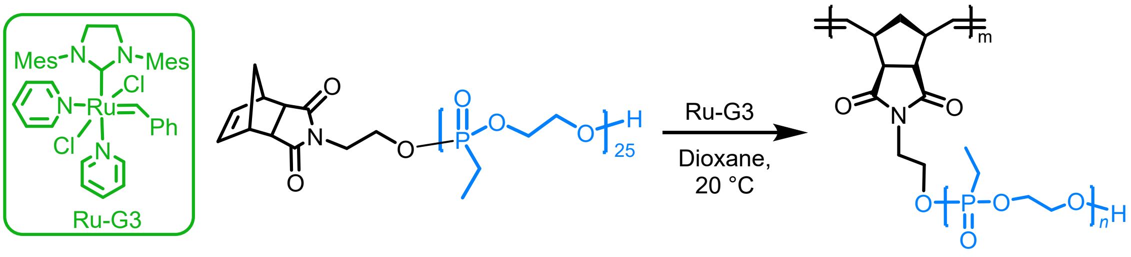

Scheme S2. Synthesis of ***bottle brush polymers*** by ring-opening metathesis polymerization of exo-norbornene polyphosphonates macroinitiator using the 3rd generation Grubbs catalyst (Ru-G3).

NOTE: During the course of this work different samples of ***bottle brush polymers*** P(NB-*g*-PetPn$_{n}$)$_{m}$ were prepared with variations in the DP_n. A representative procedure for the synthesis of a ***bottle brush polymer*** is described. Macromonomer NB-PetPn$_{25}$ (49 mg, 14 µmol, 10 eq) was dissolved in anhydrous dioxane (232 µL) in an oven-dried 2 mL vial equipped with a magnetic stirring bar. The reaction mixture was homogenized by stirring at 20 °C followed by the addition of the corresponding amount of a solution of [1,3-Dimesityl-2-imidazolidinylidene]dichloro(phenylmethylene)bis(pyridine)ruthenium(II) (Ru-G3, 0.98 mg, 1.4 µmol, 1 eq) in dioxane to give the desired [NB-PetPn$_{25}$]:[Ru-G3] molar ratio. The solution was stirred at room temperature, and depending on the experiments, samples were taken at different times before the reaction mixture was quenched by the rapid addition of an excess of ethyl vinyl ether (EVE). The resulting samples were analyzed by SEC. The crude product was purified by precipitation into cold diethyl ether (–28 °C) three times, and drying *in vacuo* to yield PetPn$_{53}$ *exo*-norbornene macromonomer as a colourless viscous liquid (0.95 g, 91 %). The molar mass was determined by SEC analyses.

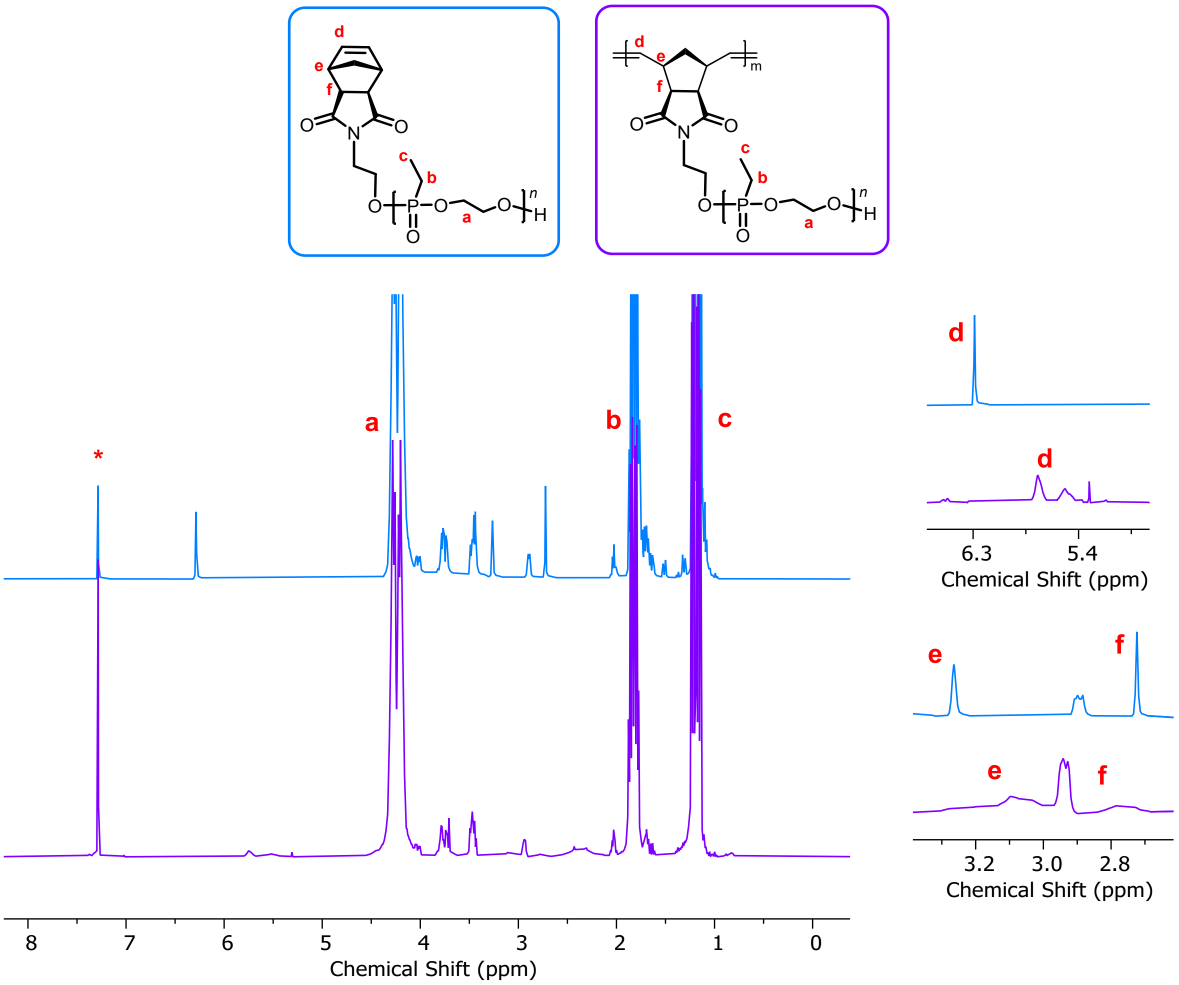

Figure S4. ^{1}H NMR (400 MHz, $CDCl_3$) spectra of NB-PEtPn$_{25}$ (blue) and the corresponding bottle-brush P(NB-*g*-PEtPn$_{25}$)$_{10}$ (purple). Deuterated solvent residual signal denoted by *.

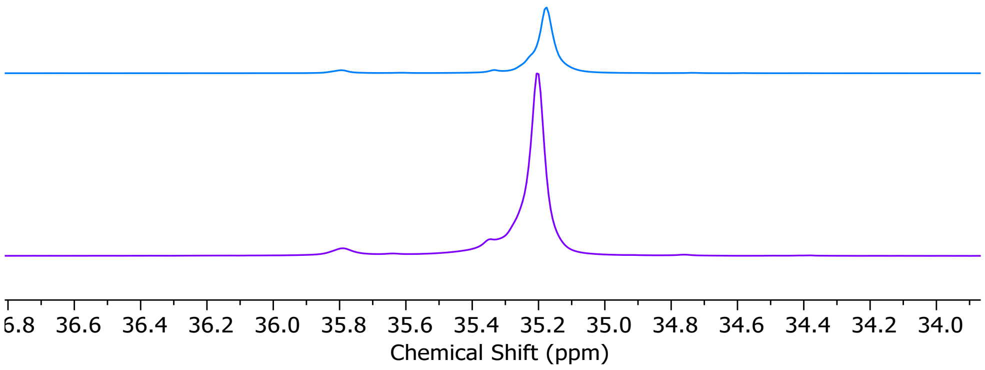

Figure S5. ^{31}P NMR (162 MHz, $CDCl_3$) spectra of NB-PEtPn$_{25}$ (blue) and the corresponding bottle-brush P(NB-*g*-PEtPn$_{25}$)$_{10}$ (purple).

Table S3. P(NB-*g*-PEtPn$_{25}$)$_n$ synthesis via ROMP of NB-PEtPn$_{25}$ varying the macromonomer to catalyst ratio.

Entry	[NB-PEtPn$_{25}$]:[Ru-G3]	Conversion[a] (%)	$M_{n\ theo}$ (kDa)	$M_{n\ SEC}$[b] (kDa)	$Đ$[b]
1	10:1	90	32.5	21.7	1.07
2	25:1	88	79.4	46.2	1.11
3	50:1	88	158.8	103.3	1.10
4	100:1	76	274.3	175.6	1.23

[a] Conversion of NB-PEtPn$_{33}$ to brush polymer is determined by integration of the peak areas of brush polymer and residual NB-PEtPn$_{33}$ from SEC measurement of the crude product.
[b] Determined from integration of the SEC signals of the high molar mass fraction (measured in DMF (0.1 M LiCl) at 60 °C vs. polystyrene standards using RI detector by conventional SEC).

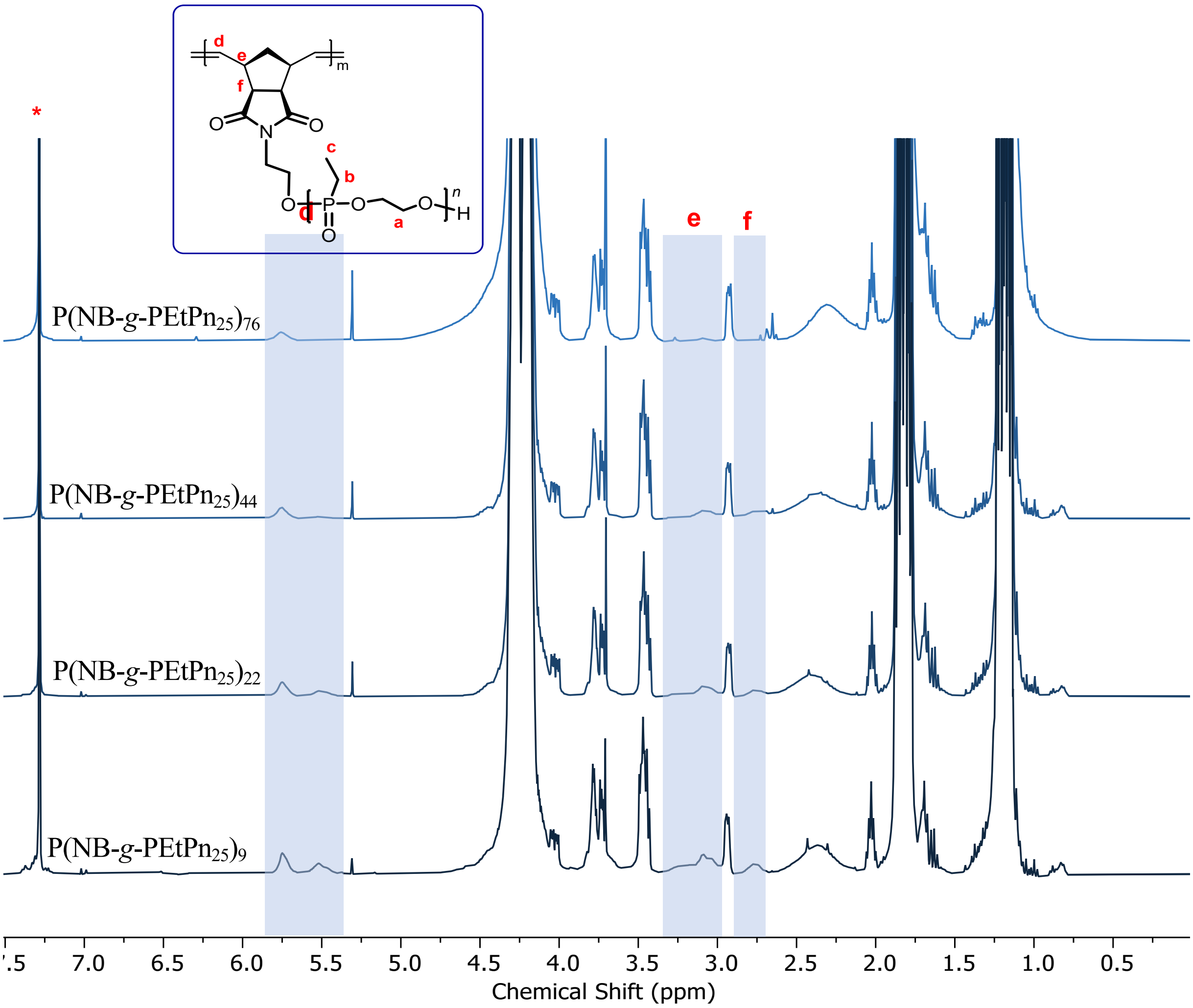

Figure S6. ^{1}H NMR (400 MHz, $CDCl_3$) spectra of bottle-brush polymers P(NB-*g*-PEtPn$_{25}$)$_m$ via ROMP with different backbone lengths. Deuterated solvent residual signal denote by *.

Table S4. P(NB-*g*-PEtPn$_n$)$_m$ synthesis via ROMP of NB-PEtPn macromonomers with varying DP_n (25, 53, 148, 263) at a macromonomer [NB-PEtPn$_n$]:[Ru-G3] ratio of 25:1.

Entry	Macromonomer	[NB-PEtPn$_n$]: [Ru-G3]	Conversion (%)[a]	$M_{n\ SEC}$ [b] (kDa)	$Đ$ [b]
1	NB-PEtPn$_{25}$	25:1	88	46.2	1.11
2	NB-PEtPn$_{53}$	25:1	75	66.8	1.09
3	NB-PEtPn$_{148}$	25:1	58	144.2	1.08
4	NB-PEtPn$_{263}$	25:1	--	---	---

[a] Conversion of NB-PEtPn$_{33}$ to brush polymer is determined by integration of the peak areas of brush polymer and residual NB-PEtPn$_{33}$ from SEC measurement of the crude product.
[b] Determined from integration of the SEC signals of the high molar mass fraction (measured in DMF (0.1 M LiCl) at 60 °C vs. polystyrene standards using RI detector by conventional SEC).

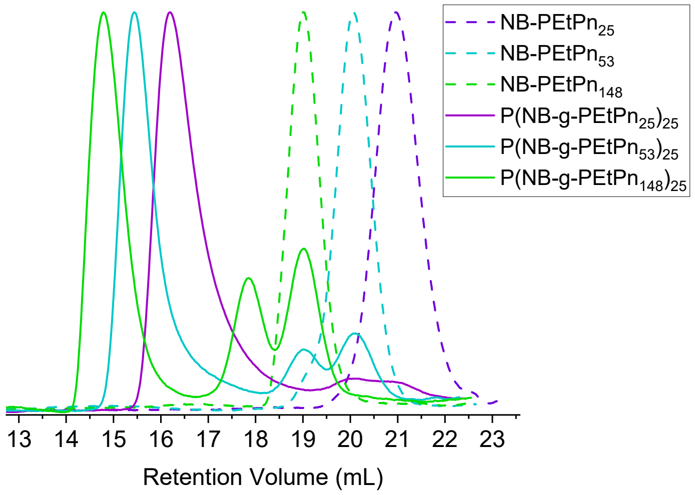

Figure S7. SEC elugrams (normalized RI) of bottle-brush polymers P(NB-*g*-PEtPn$_{25}$)$_m$ (solid lines) via ROMPs with varying DP_n (25, 53, 148) of NB-PEtPn macromonomers (dotted lines) with a [NB-PEtPn$_n$]:[Ru-G3] ratio of 25:1 (measured in DMF (0.1 M LiCl) at 60 °C vs. polystyrene standards using RI detector by conventional SEC)).

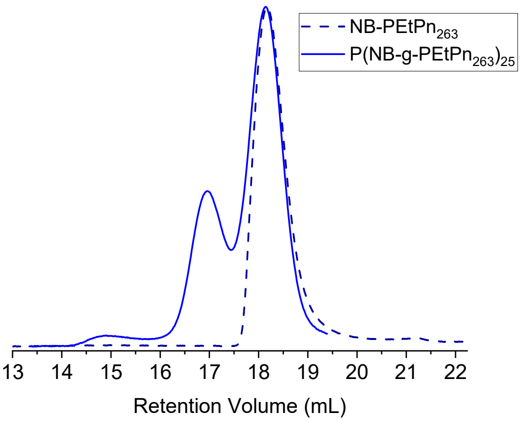

Figure S8. SEC elugrams (2 mg mL^{-1}) (normalized RI) of bottle-brush polymers P(NB-*g*-PEtPn$_{263}$)$_m$ (solid lines) via ROMPs with DP_n (263) of the NB-PEtPn macromonomer (dotted lines) with a [NB-PEtPn$_n$]:[Ru-G3] ratio of 25:1 (measured in DMF (0.1 M LiCl) at 60 °C vs. polystyrene standards using RI detector by conventional SEC).

Synthesis of bottle-brush polymers in aqueous media

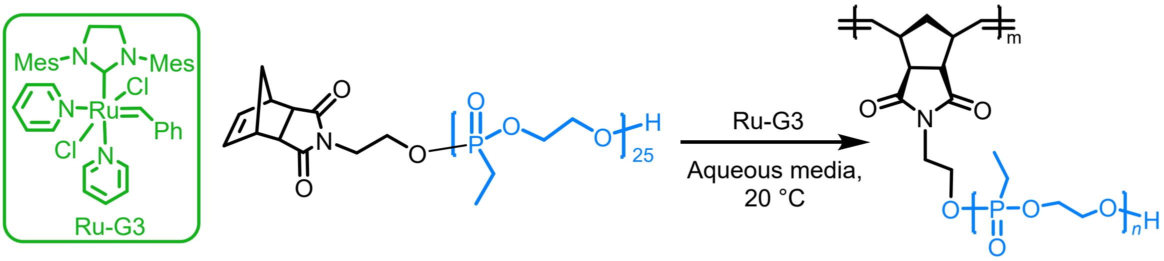

Scheme S3. Synthesis of ***bottle brush polymers*** by ring-opening metathesis polymerization of *exo*-norbornene polyphosphonates macroinitiator using the 3rd generation Grubbs catalyst (Ru-G3) in aqueous media.

NOTE: During the course of this work different samples of ***bottle brush polymers*** were prepared with variations of the aqueous media:

1) 9:1 v/v H_2O/THF (100 mM Na_2HPO_4) (pH = 2 with HCl)
2) 9:1 v/v H_2O/THF (pH = 2 with HCl)
3) 9:1 v/v H_2O/THF (100 mM NaCl)

A representative procedure for the synthesis of a ***bottle brush polymer*** in aqueous media is described.

Macromonomer PetPn$_{33}$ (27 mg, 5.66 µmol, 50 eq) was dissolved in 9:1 v/v H_2O/THF (100 mM Na_2HPO_4) (pH = 2 with HCl) (1.7 mL) in a 2 mL vial equipped with a magnetic stirring bar. The reaction mixture was homogenized by stirring at 20 °C followed by the addition of the corresponding amount of a solution of [1,3-Dimesityl-2-imidazolidinylidene]dichloro(phenylmethylene) bis(pyridine)

ruthenium(II) (Ru-G3, 0.08 mg, 0.113 □mol, 1 eq) in THF (3.5 mg mL^{-1}) to give the desired a [NB-PetPn$_{33}$]:[Ru-G3] ratio. The solution was stirred at room temperature for 2h before the reaction mixture was quenched by the rapid addition of an excess of diethylene glycol vinyl ether (DGVE). The resulting samples were analyzed by SEC.

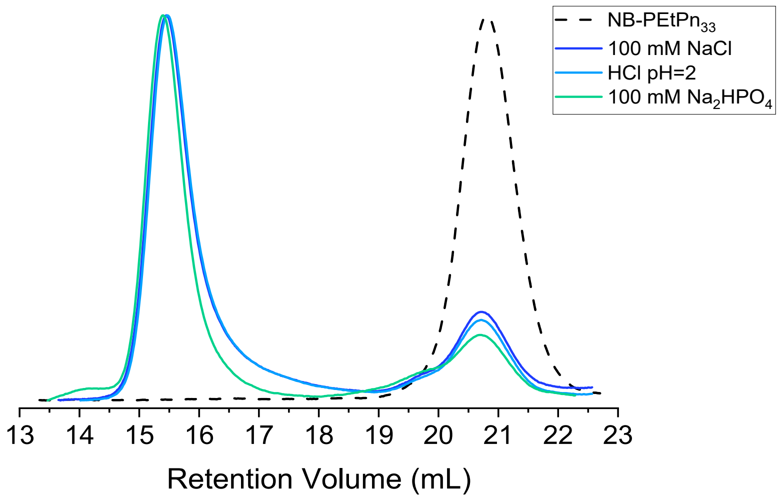

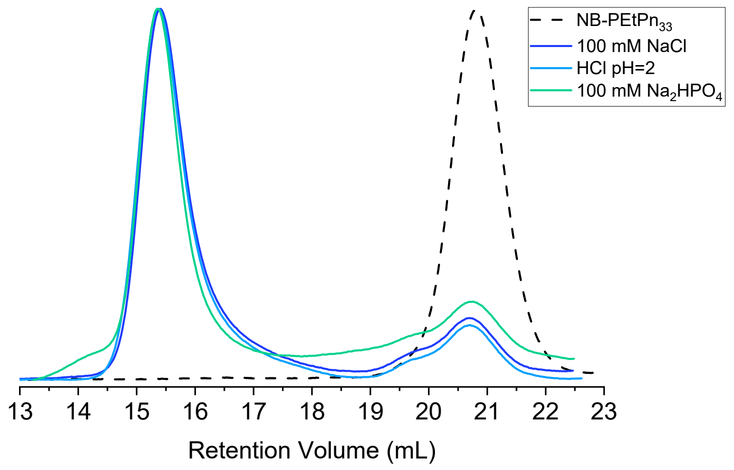

Figure S9**.** SEC elugrams (normalized RI) of bottle-brush polymers P(NB-*g*-PEtPn$_{33}$)$_m$ (solid lines) via ROMPs of NB-PEtPn$_{33}$ macromonomers (dotted line) with a [NB-PEtPn$_n$]:[Ru-G3] ratio of 50:1 under different aqueous media conditions after 3h (above) and 18 h (below) at a 10 mg mL^{-1} concentration (measured in DMF (0.1 M LiCl) at 60 °C vs. polystyrene standards using RI detector by conventional SEC).

Synthesis of double hydrophilic block copolymers in aqueous media

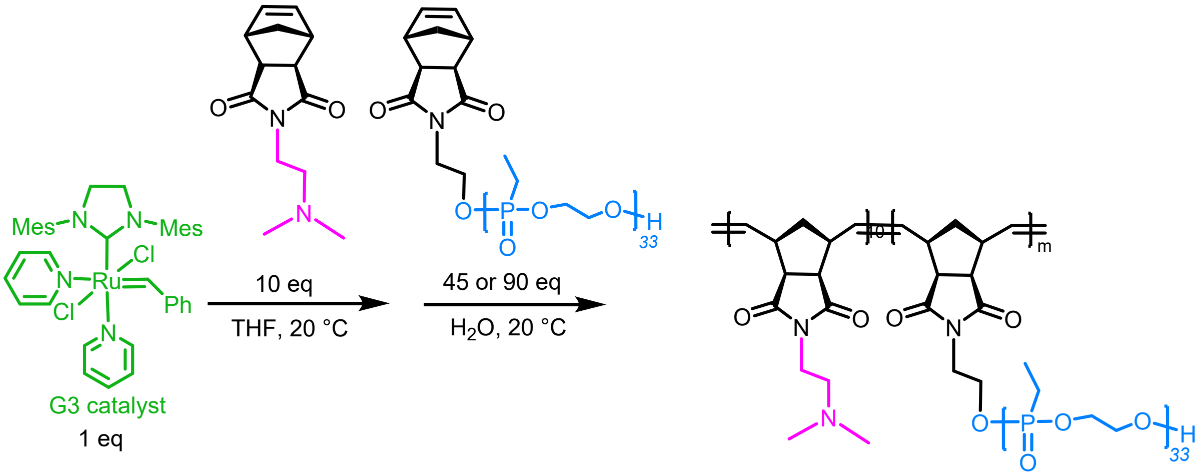

Scheme S4. Synthesis of ***double hydrophilic block copolymers*** by ring-opening metathesis polymerization of *exo*-norbornene polyphosphonates via of a second macroinitiator using the 3rd generation Grubbs catalyst in aqueous conditions.

Macromonomer NB-PEtPn$_{33}$ (100 mg, 21.3 μmol) was dissolved in 1.0 mL of a mixture of 9:1 *v/v* [H_2O (100 mM NaCl):THF] in a 2 mL vial equipped with a magnetic stirring bar and the reaction mixture was homogenized by stirring at 20 °C. Then, in an separate 2 mL vial, NB-Nme$_2$ (10.4 mg, 42.7 μmol) was dissolved in 100 μL THF, followed by the addition of the Ru-G3 catalyst (3.1 mg, 4.3 μmol) in THF (100 μL) to give the desired [NB-PetPn$_{33}$]:[Ru-G3] ratio of 10:1. After stirring for 3 min, 5 μL and 10 μL of the P[(NB-*g*-Nme$_2$)$_n$] macromonomer were added to two separate 2 mL vials equipped with a magnetic stirring bar, and diluted to a final 50 μL volume of THF, respectively. Finally, 450 μL of NB-PetPn$_{33}$ aqueous solution was added to each one of these vials, and stirred at room temperature for 2h before the reaction mixture was quenched by the rapid addition of an excess of diethylene glycol vinyl ether (DGVE). The resulting samples were analyzed by SEC.

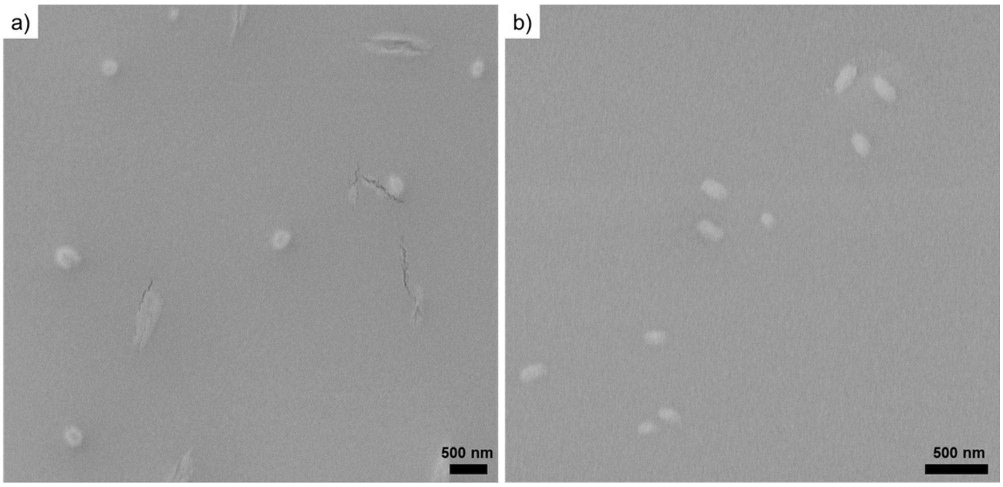

Figure S10: SEM measurement of a) P[(NB-NMe_2)$_{10}$–*b*–(NB-*g*-$PEtP_{33}$)$_{45}$] and b) P[(NB-NMe_2)$_{10}$–*b*–(NB-*g*-$PEtP_{33}$)$_{90}$] BBPs sputtered with 3 nm gold. Scale bar = 500 nm.

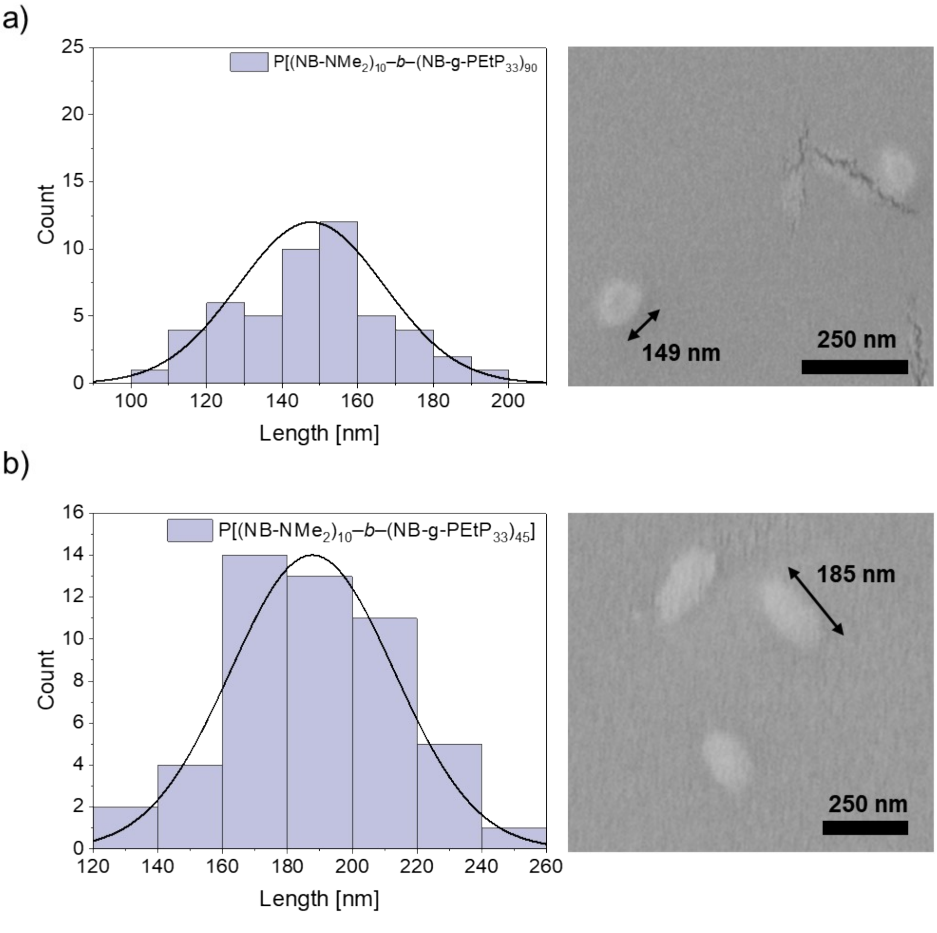

Figure S11: Distribution function of a) P[(NB-NMe_2)$_{10}$–*b*–(NB-g-$PEtP_{33}$)$_{45}$] and b) P[(NB-NMe_2)$_{10}$–*b*–(NB-g-$PEtP_{33}$)$_{90}$] consisting of 50 counts and the corresponding SEM zoom ins with the indication of average length. Sputtered with 3 nm gold. Scalebar = 250 nm.

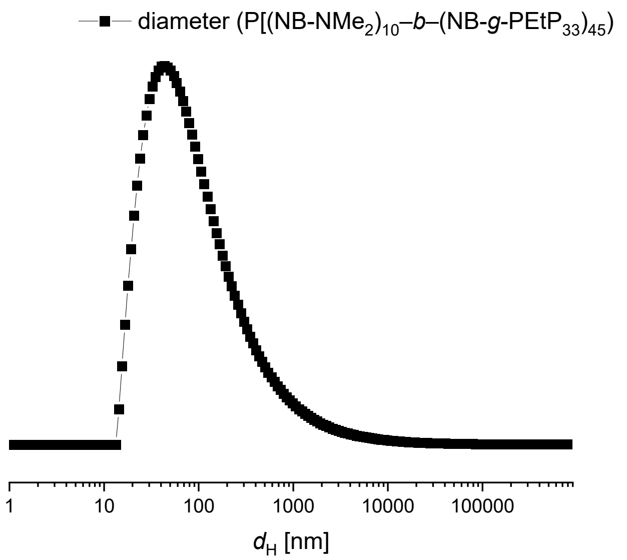

Figure S12: The dynamic size distribution of (P[(NB-NMe$_2$)$_{10}$–*b*–(NB-*g*-PEtP$_{33}$)$_{45}$), was measured in DMF with a concentration of 1 mg mL^{-1}.

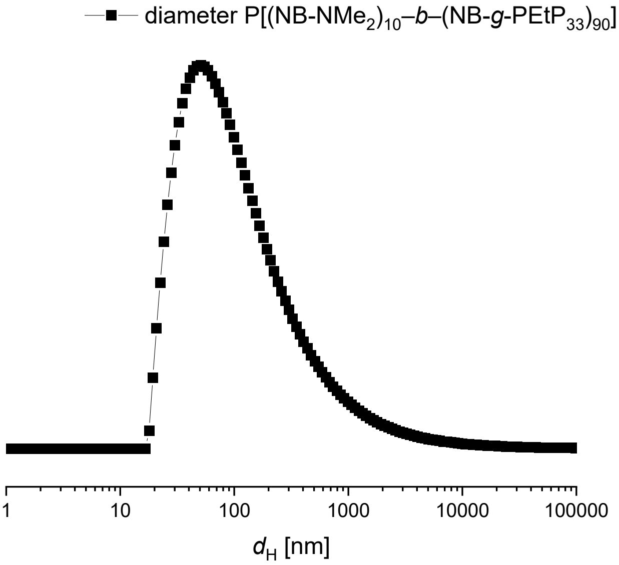

Figure S13: The dynamic size distribution of (P[(NB-NMe$_2$)$_{10}$–*b*–(NB-*g*-PEtP$_{33}$)$_{90}$), was measured in DMF with a concentration of 1 mg mL^{-1}.

Degradation studies

Macromonomer (P[(NB-NMe$_2$)$_{10}$–*b*–(NB-*g*-PEtP$_{33}$)$_{45}$) (5 mg) was dissolved in 1.5 mL of a buffer solution (pH = 11) in a 2 mL vial equipped with a magnetic stirring bar and the reaction mixture was stirred at 20 °C for 24 h. The reaction mixture was freeze-dried, and the sample was dissolved in $CHCl_3$, and filtered to eliminate the excess of insoluble substances. The resulting samples were analyzed by SEC and NMR spectroscopy.

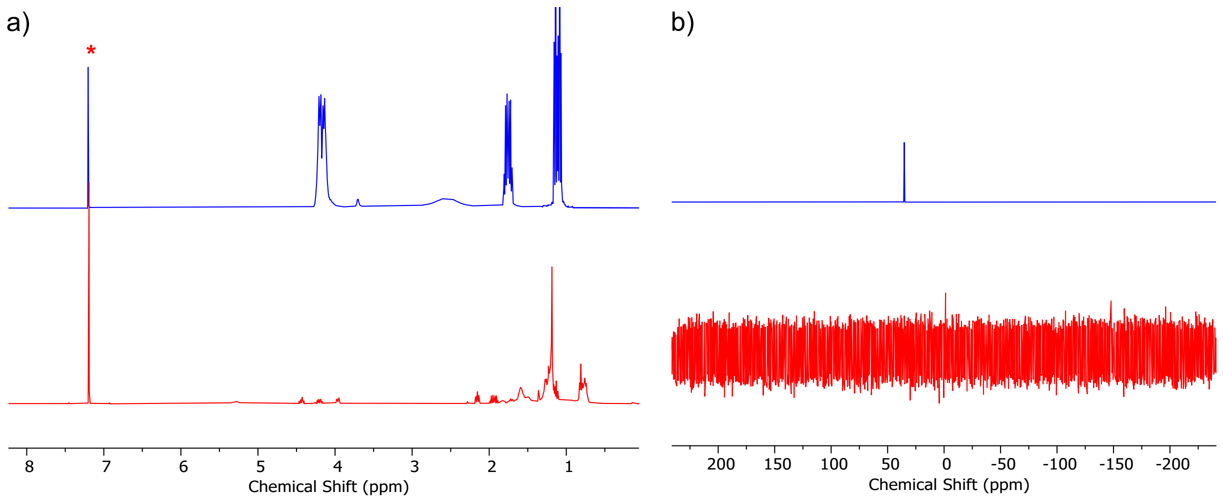

Figure S13**.** ^{1}H NMR (left) and ^{31}P NMR (right) (400/ 162 MHz, $CDCl_3$) spectra of (P[(NB-NMe$_2$)$_{10}$–*b*–(NB-*g*-PEtP$_{33}$)$_{45}$ (blue) and the corresponding degradation product (red). Deuterated solvent residual signal denoted by *.

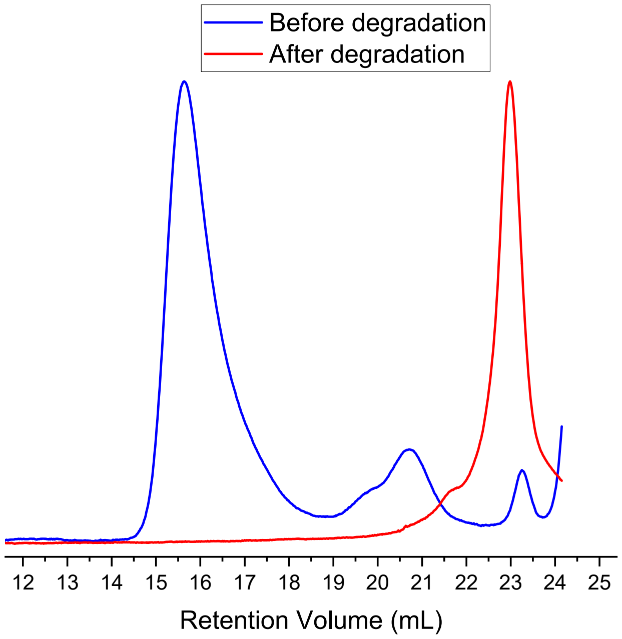

Figure S14**.** SEC elugrams (normalized RI) of bottle-brush polymers (P[(NB-NMe$_2$)$_{10}$–*b*–(NB-*g*-PEtP$_{33}$)$_{45}$ (blue line) (M_n = 71.9 kDa, *Đ* = 1.12) mL^{-1} and the corresponding degradation product (red line) (M_n = 0.5 kDa, *Đ* = 1.20) (measured in DMF (0.1 M LiCl) at 60 °C vs. polystyrene standards using RI detector).

References

S1. K. E. I. Kluthe, M. Wagner and M. Klapper, *Macromolecules*, 2021, **54**, 1465-1477.

S2. T. Wolf, T. Steinbach and F. R. Wurm, *Macromolecules*, 2015, **48**, 3853-3863.

S3. S. C. Radzinski, J. C. Foster, S. J. Scannelli, J. R. Weaver, K. J. Arrington and J. B. Matson, *ACS Macro Letters*, 2017, **6**, 1175-1179.

S4. J. L. Hutter and J. Bechhoefer, *Rev. Sci. Instrum.*, 1993, **64**, 1868-1873.

S5. N. Hampu, J. R. Werber and M. A. Hillmyer, *ACS Applied Materials & Interfaces*, 2020, **12**, 45351-45362.
